# Supplementary material for: Soybean root nodule occupancy: competition between Bradyrhizobium and Sinorhizobium strains inoculated at different plant growth stages
Source: Appl Environ Microbiol. 2026 Feb 9;92(3):e02489-25. doi: 10.1128/aem.02489-25 (PMC12997764; doi:10.1128/aem.02489-25)

Revised manuscript: AEM02160-25
Applied and Environmental Microbiology
Authors: Knoll M and Mirza B

Supplemental Material For

Soybean Root Nodule Occupancy: Competition Between *Bradyrhizobium* and *Sinorhizobium* Strains Inoculated at Different Plant Growth Stages

Matthew Knoll[1], Babur S Mirza[1*]

[1] Department of Biology, Missouri State University, Springfield, Missouri, 65897, USA

*Corresponding author: Babur S Mirza
Missouri State University
Department of Biology
901 S. National Ave. Springfield, MO 65897
Phone: 417–836–5062
Fax: 417–836–4204
E-mail: baburmirza@missouristate.edu

Table S1: Distribution of bacterial sequences belonging to various genera in the rhizosphere of soybean (*Glycine max*) plants inoculated with *Bradyrhizobium diazoefficiens* USDA 110 and *Sinorhizobium fredii* USDA 191 at three inoculation time points (T_0, T_2, and T_4) and at varying BR:SR inoculum ratios (1:1, 1:100, and 100:1). In addition to sequences related to other bacterial genera, the table reports the average BR and SR sequence abundances within the rhizosphere, the percentage distribution of BR and SR sequences (% BR:SR Seqs), and the adjusted percentages accounting for three 16S rRNA gene copies per *Sinorhizobium* cell (Adj. % BR:SR Seq). Values are reported as means with standard error (±SE).

Inoculation Time	BR:SR Ratio	Plants (n)	Total Seqs	% BR:SR Seqs	Adj. % BR:SR Seq	BR Seqs (Avg.)	SR Seqs (Avg.)	*Bacillus* spp. Seqs (Avg.)	*Comamonas* spp. Seqs (Avg.)	*Geobacter* spp. Seqs (Avg.)
T0	1:1	7	8,103	24: 76	49: 51	281±55	877±184	18,863±6,608	4,412±3,719	1,197±794
	1:100	7	10,397	38: 62	65: 35	565±281	921±317	15002±3261	862±215	958±554
	100:1	8	8,159	52: 48	77: 23	534±125	486±74	13,101±2,904	415±125	684±413
T2	1:1	9	1,3224	57: 43	80: 20	840±492	629±122	12,058±2,187	1,760±330	5,462±2,439
	1:100	6	5,286	31: 69	57: 43	273±58	608±144	9,959±1,638	3,952±3,062	844±290
	100:1	9	5,990	37: 63	64: 36	142±47	421±111	11,463±1,414	1,190±432	2,271±1,038
T4	1:1	9	4,959	37: 63	64: 36	205±31	346±51	8,947±3,123	404±104	532±135
	1:100	7	5,349	56:44	79: 21	338±205	426±259	2,590±446	329±99	748±486
	100:1	9	6,425	53: 47	77: 23	380±171	334±143	4,585±1326	383±76	815±446

Table S2: Colony-forming unit (CFU) determination for bacterial cultures. The table shows CFU counts of harvested rhizobial cells obtained from individual plates, corresponding dilution factors, and calculated CFU per 100 µL for each culture. Colony forming units per 100 µL.

Plate	Strain	T0 Plates	Dilution	T0 CFU/100 µL	T2 Plates	Dilution	T2 CFU/100 µL	T4 Plates	Dilution	T4 CFU/100 µL
1	BR	191	10^{-5}	1.91E+07	150	10^{-5}	1.50E+07	132	10^{-5}	1.32E+07
2	BR	33	10^{-6}	3.30E+07	43	10^{-6}	4.30E+07	38	10^{-6}	3.80E+07
3	BR	ND	ND	ND	135	10^{-5}	1.35E+07	136	10^{-5}	1.36E+07
4	BR	138	10^{-5}	1.38E+07	ND	ND	ND	42	10^{-6}	4.20E+07
5	BR	38	10^{-6}	3.80E+07	166	10^{-5}	1.66E+07	109	10^{-5}	1.09E+07
6	BR	178	10^{-5}	1.78E+07	34	10^{-6}	3.40E+07	35	10^{-6}	3.50E+07
7	BR	147	10^{-5}	1.47E+07	130	10^{-5}	1.30E+07	127	10^{-5}	1.27E+07
8	BR	36	10^{-6}	3.60E+07	38	10^{-6}	3.80E+07	44	10^{-6}	4.40E+07
9	BR	169	10^{-5}	1.69E+07	138	10^{-5}	1.38E+07	92	10^{-5}	9.20E+06
**BR Mean (±SE)**				2.38×10^7 ($\pm 0.36 \times 10^7$)			2.34×10^7 ($\pm 0.45 \times 10^7$)			2.43×10^7 ($\pm 0.54 \times 10^7$SE)
1	SR	32	10^{-6}	3.20E+07	86	10^{-5}	8.60E+06	31	10^{-6}	3.10E+07
2	SR	48	10^{-6}	4.80E+07	41	10^{-6}	4.10E+07	33	10^{-6}	3.30E+07
3	SR	105	10^{-5}	1.05E+07	93	10^{-5}	9.30E+06	35	10^{-6}	3.50E+07
4	SR	35	10^{-6}	3.50E+07	99	10^{-5}	9.90E+06	71	10^{-5}	7.10E+06
5	SR	30	10^{-6}	3.00E+07	40	10^{-6}	4.00E+07	37	10^{-6}	3.70E+07
6	SR	87	10^{-5}	8.70E+06	35	10^{-6}	3.50E+07	74	10^{-5}	7.40E+06
7	SR	ND	ND	ND	42	10^{-6}	4.20E+07	36	10^{-6}	3.60E+07
8	SR	101	10^{-5}	1.01E+07	114	10^{-5}	1.14E+07	66	10^{-5}	6.60E+06
9	SR	95	10^{-5}	9.50E+06	59	10^{-5}	5.90E+06	ND	ND	ND
**SR Mean (±SE)**				2.30×10^7 ($\pm 0.53 \times 10^7$)			2.40×10^7 ($\pm 0.69 \times 10^7$)			2.41×10^7 ($\pm 0.51 \times 10^7$)

ND indicates samples that could not be quantified because of plate contamination or lack of observable growth.

Fig S1. Adjusted relative distribution of *Bradyrhizobium diazoefficiens* USDA 110 (BR) and *Sinorhizobium fredii* USDA 191 (SR) cells within soybean root nodules from 24 control plants inoculated with either BR or SR strain at three time points: sowing (T_0), two weeks (T_2), and four weeks (T_4) after planting. A total of 3.74 million sequences were analyzed from 24 plants, with 10 nodules randomly selected per plant. To better estimate relative cell abundances, SR sequence counts were adjusted to account for the three copies of the 16S rRNA gene per cell, whereas BR contains only a single copy.

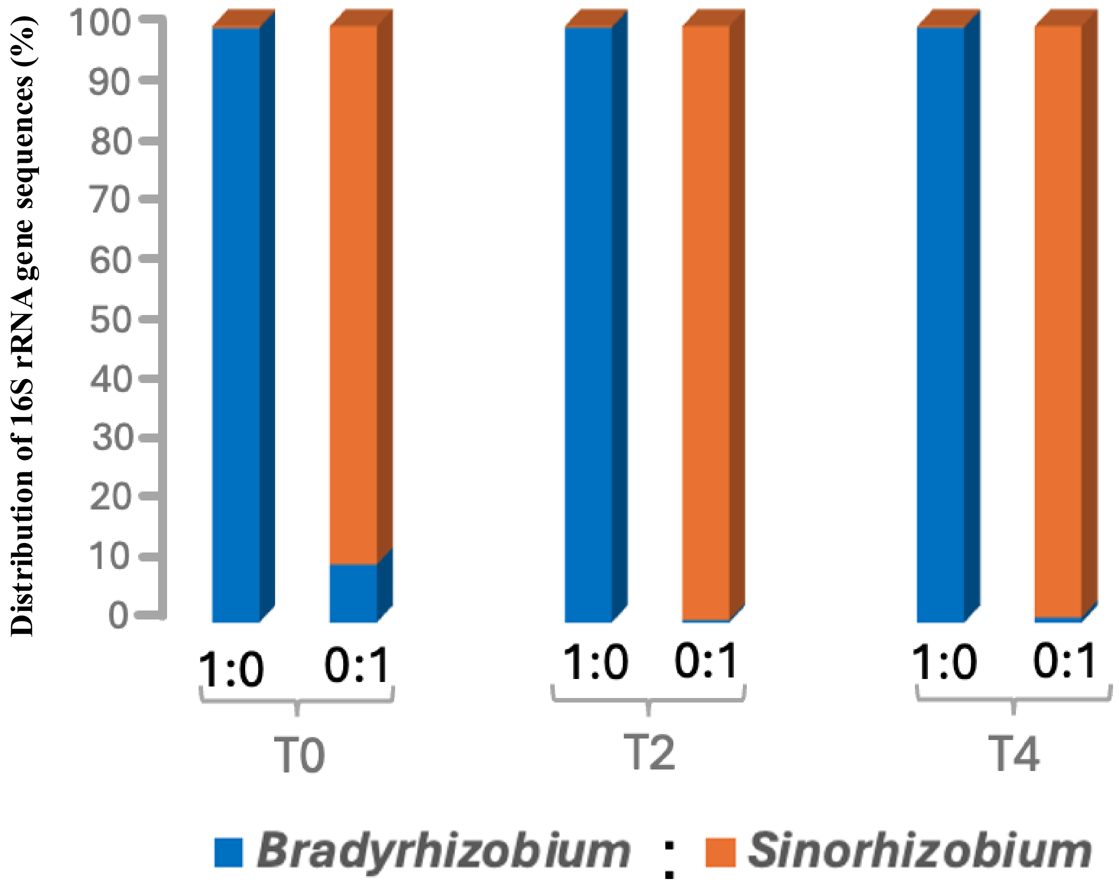

Fig S2: Log-transformed copy numbers of 16S rRNA genes (mean ± standard error) showing the relative distribution of *Bradyrhizobium diazoefficiens* USDA 110 (BR) and *Sinorhizobium fredii* USDA 191 (SR) within soybean root nodules of 71 plants co-inoculated with both strains. Plants were inoculated at three time points—sowing (T_0), two weeks (T_2), and four weeks (T_4) after germination—using three BR:SR inoculation ratios (1:1, 1:100, and 100:1). A total of 3.94 million sequences were analyzed from nodules (10 per plant).

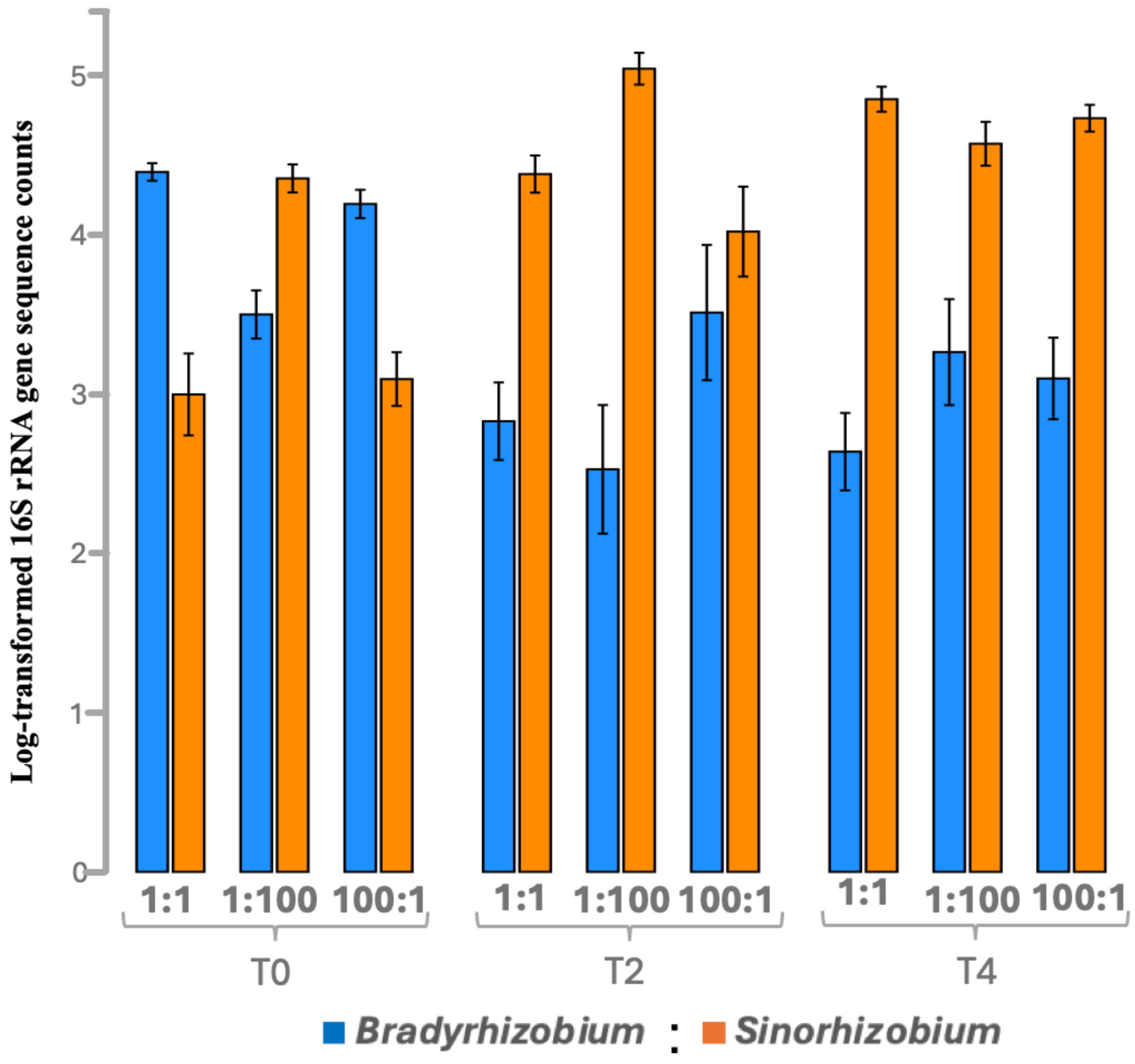

Fig. S3. Adjusted relative distribution of *Bradyrhizobium diazoefficiens* USDA 110 (BR) and *Sinorhizobium fredii* USDA 191 (SR) cells within soybean root nodules of 71 plants co-inoculated with both strains at three time points: sowing (T_0), two weeks (T_2), and four weeks (T_4) after germination, using three BR:SR inoculation ratios (1:1, 1:100, and 100:1). A total of 3.94 million sequences were analyzed from nodules (10 per plant). To estimate relative cell abundances, SR sequence counts were adjusted for the presence of three copies of the 16S rRNA gene per cell, whereas BR contains only a single copy.

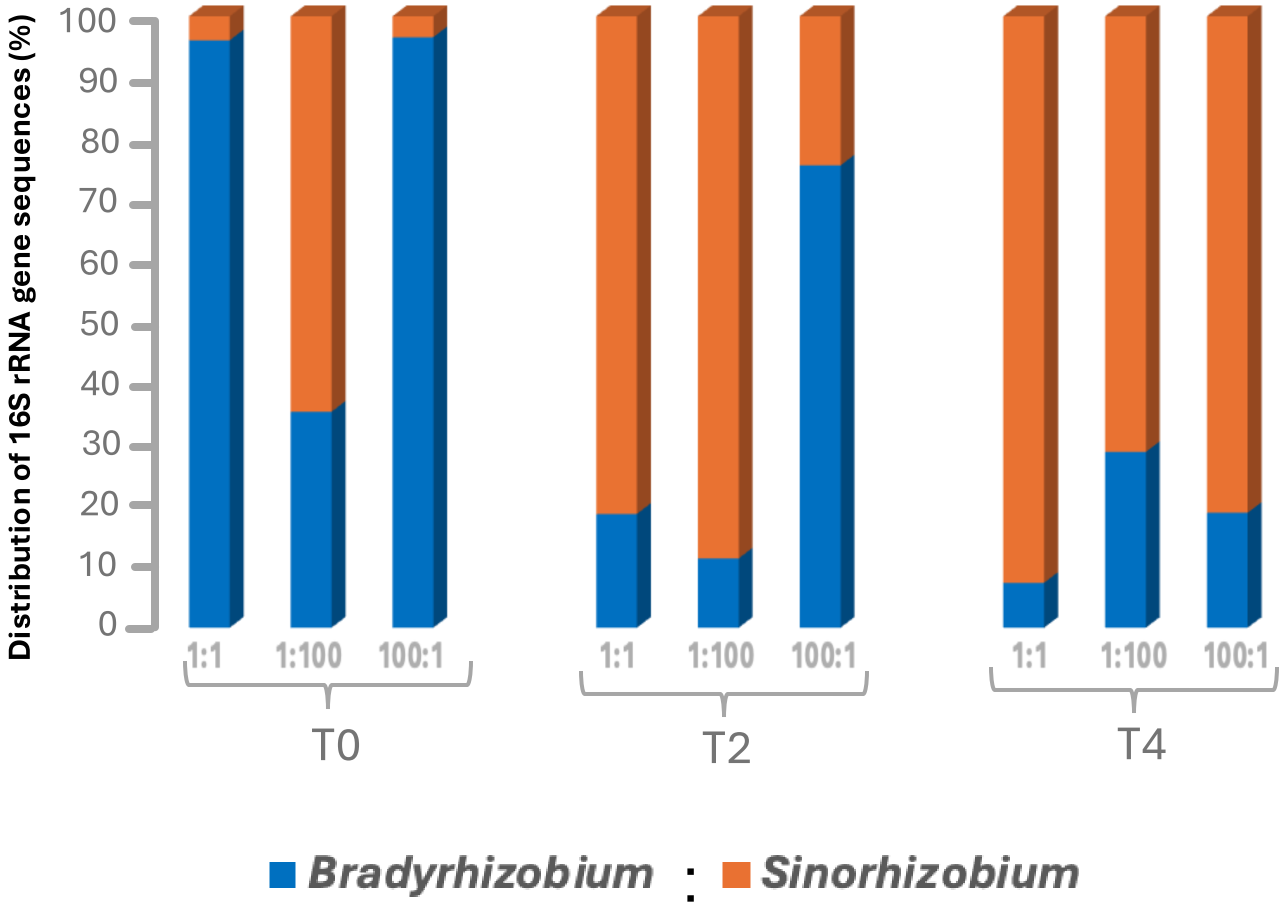

Fig. S4: Adjusted log-transformed copy numbers of 16S rRNA genes (mean ± standard error) representing the relative distribution of *Bradyrhizobium diazoefficiens* USDA 110 (BR) and *Sinorhizobium fredii* USDA 191 (SR) within soybean root nodules of 71 plants co-inoculated with both strains. SR values were adjusted to account for three copies of the 16S rRNA gene per cell, whereas BR carries a single copy. Plants were inoculated at sowing (T_0), two weeks (T_2), and four weeks (T_4) after germination using three BR:SR inoculation ratios (1:1, 1:100, and 100:1). A total of 3.94 million sequences were analyzed from nodules (10 per plant).

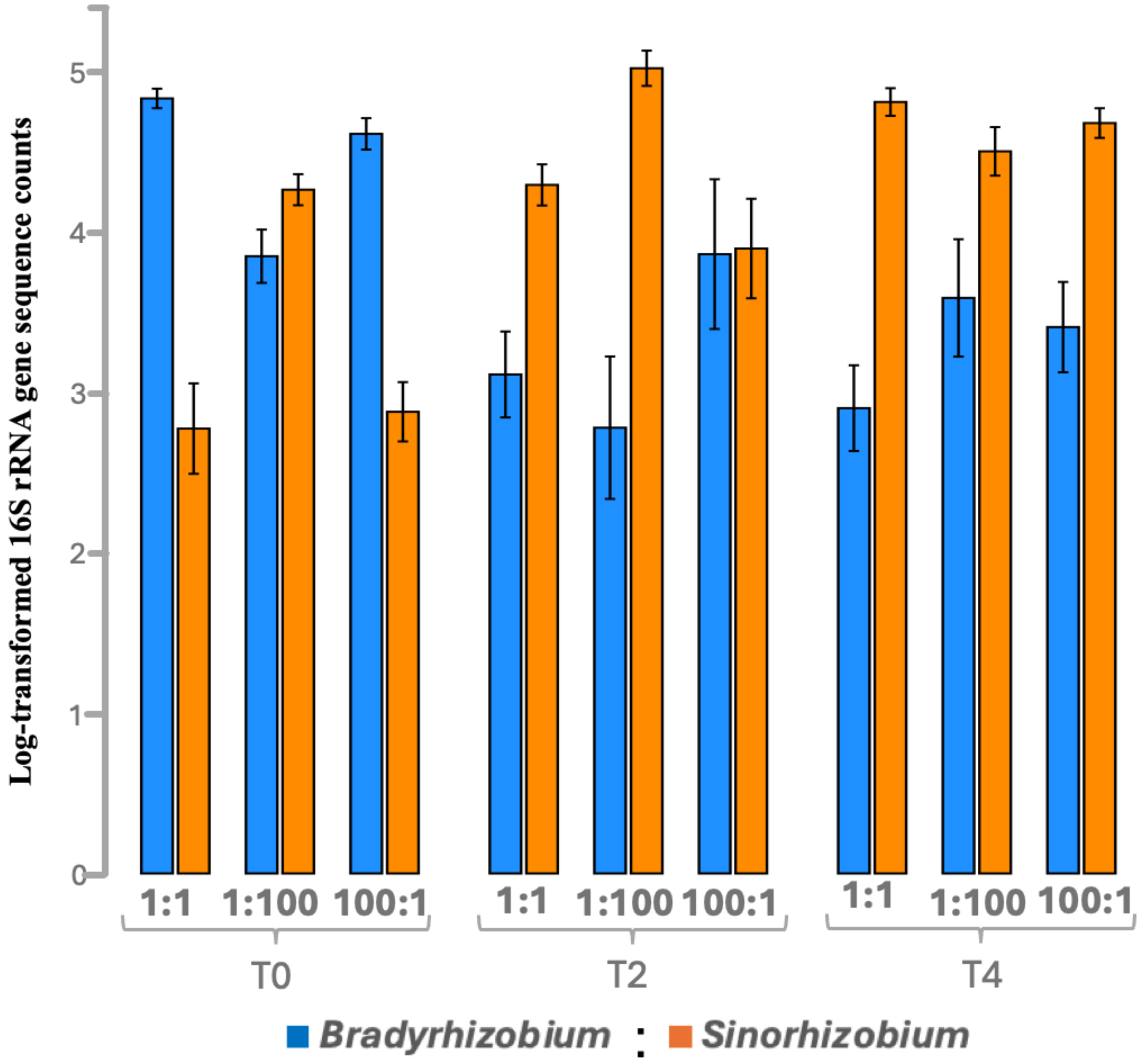

Fig. S5: Adjusted relative distribution of *Bradyrhizobium diazoefficiens* USDA 110 (BR) and *Sinorhizobium fredii* USDA 191 (SR) cells in the rhizosphere of soybean plants at harvest (day 56). Rhizosphere samples were collected from 71 plants co-inoculated with both strains at three inoculation time points: sowing (T_0), two weeks (T_2), and four weeks (T_4) after germination, using BR:SR ratios of 1:1, 1:100, and 100:1. To estimate relative cell abundances, SR sequence counts were adjusted for three copies of the 16S rRNA gene per cell, whereas BR contains only a single copy.

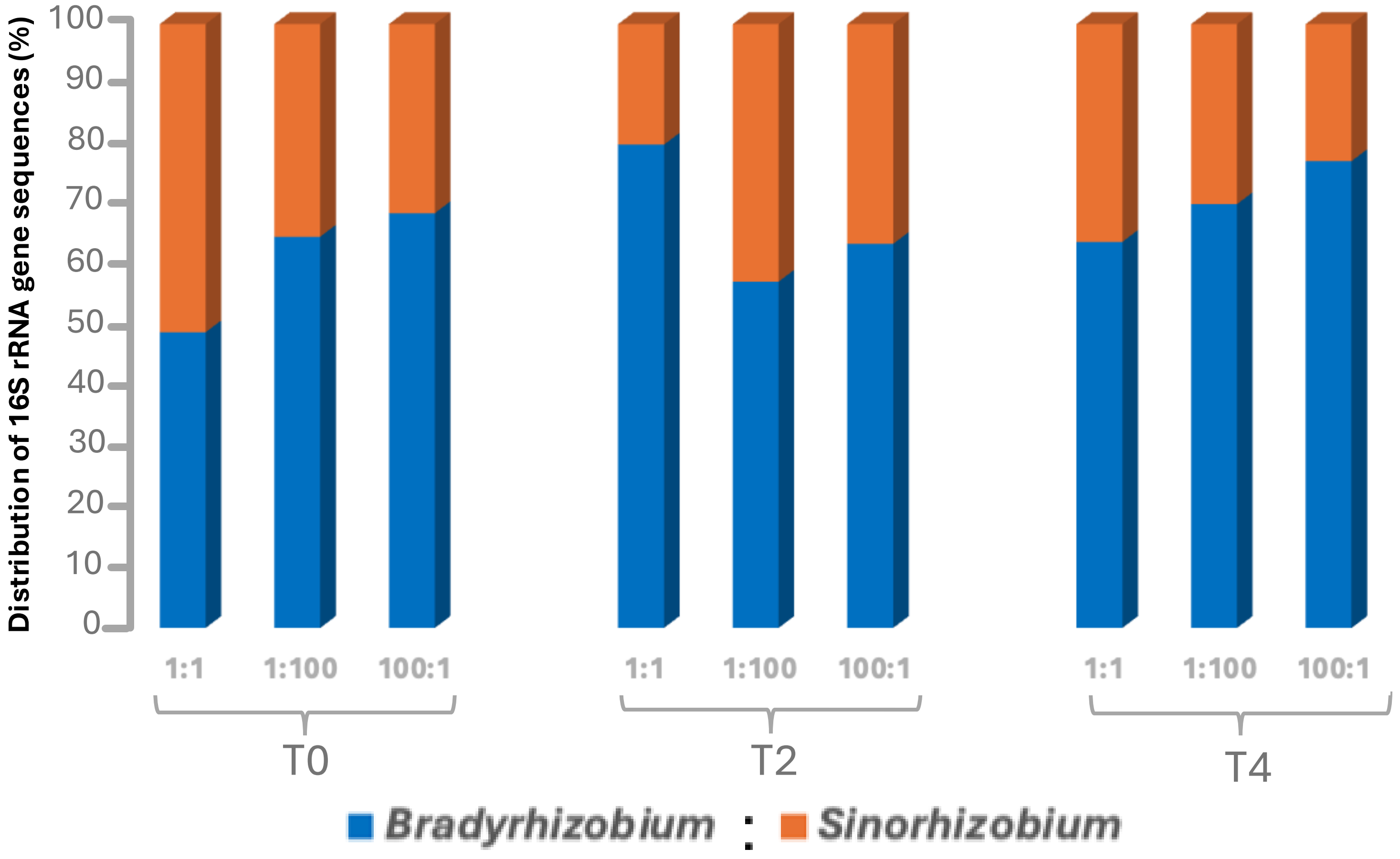

Fig. S6 AB: A) NMDS plot illustrating the distribution of *Bradyrhizobium diazoefficiens* (BR) and *Sinorhizobium fredii* (SR) in the rhizosphere of soybean plants. Plants were inoculated with three different BR:SR cell ratios (1:1, 100:1, and 1:100) and sampled at three time points. No significant effect of inoculation ratio or timing was observed on the abundance or distribution of BR and SR.
B) NMDS plot showing the distribution of different bacterial species including BR and SR detected in the rhizosphere of the same soybean plants under the same inoculation ratios and sampling times. Similarly, no significant effect of inoculation ratio or timing was observed on the overall bacterial community structure.

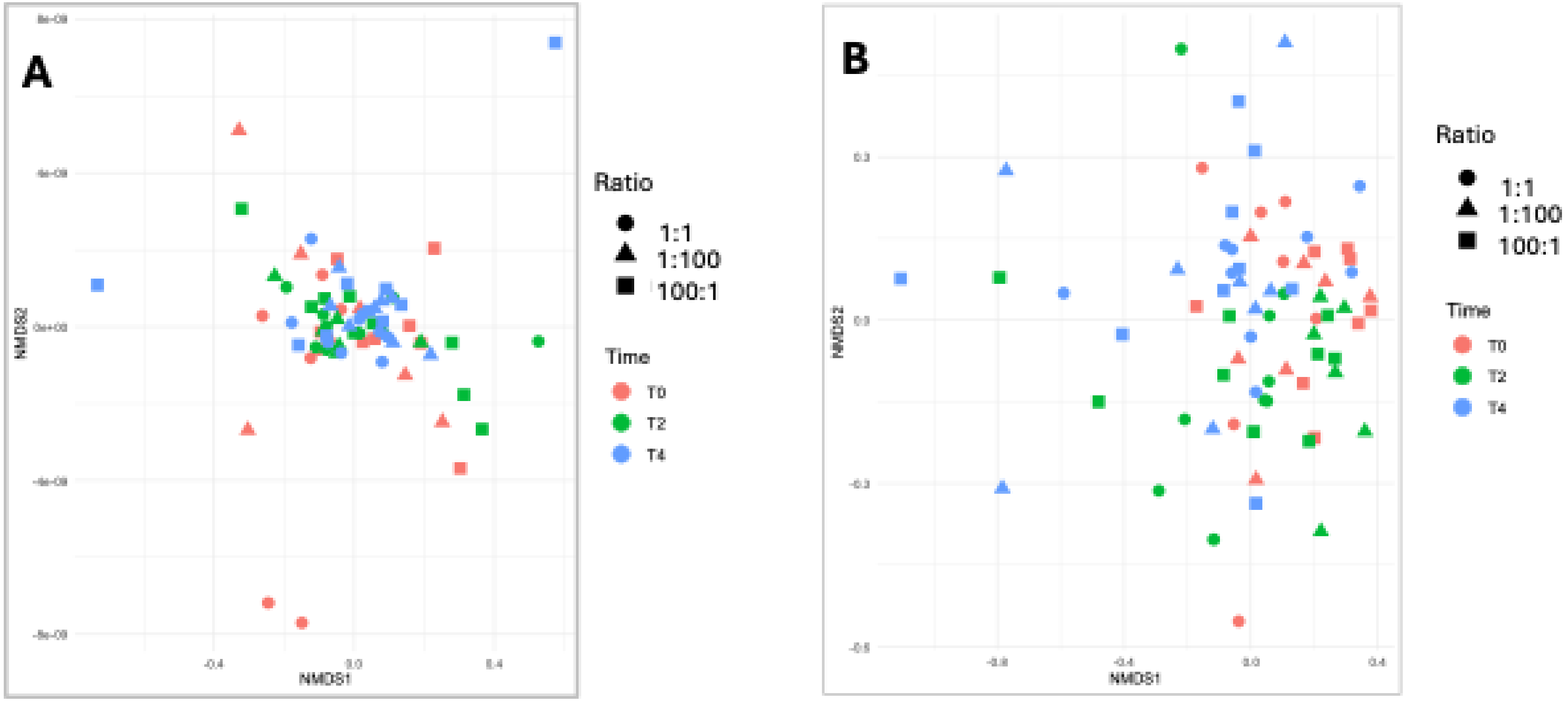

Fig. S7: Bar graphs showing diversity metrics (Species richness, Shannon diversity, and Simpson diversity) of the rhizosphere bacterial community in soybean plants inoculated with different BR:SR cell ratios (1:1, 100:1, 1:100) at three time points (T0, T2, T4). Bars represent mean values, and error bars indicate standard error. No consistent effect of inoculation ratio or timing was observed on overall bacterial diversity.

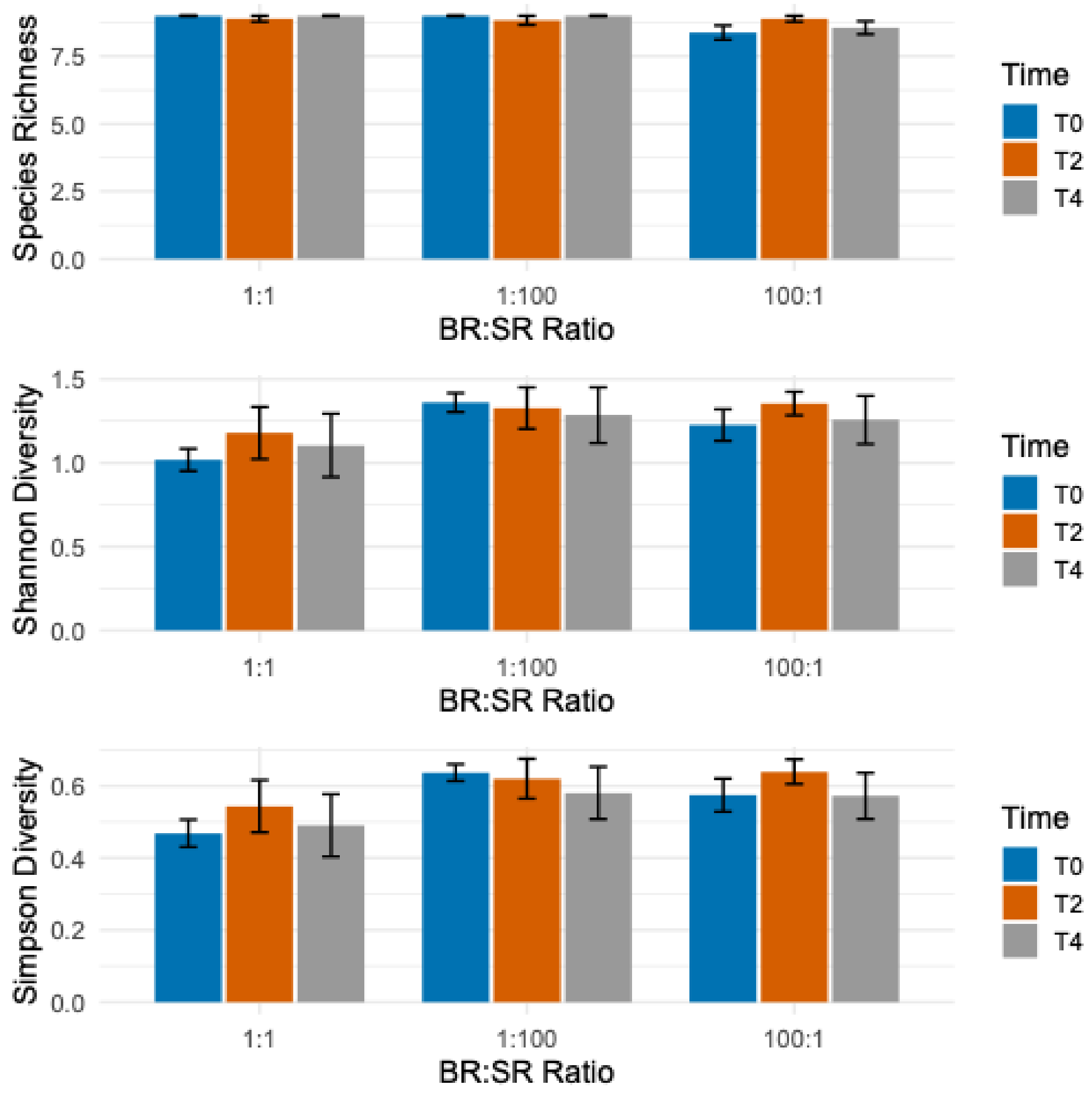

Fig. S8: Average number of nodules per plant with standard error bars. Different letters above the bars indicate statistically significant differences based on Tukey's HSD test; bars sharing the same letter are not significantly different. A total of 720 root nodules were collected by sampling 10 nodules from each of 72 plants.

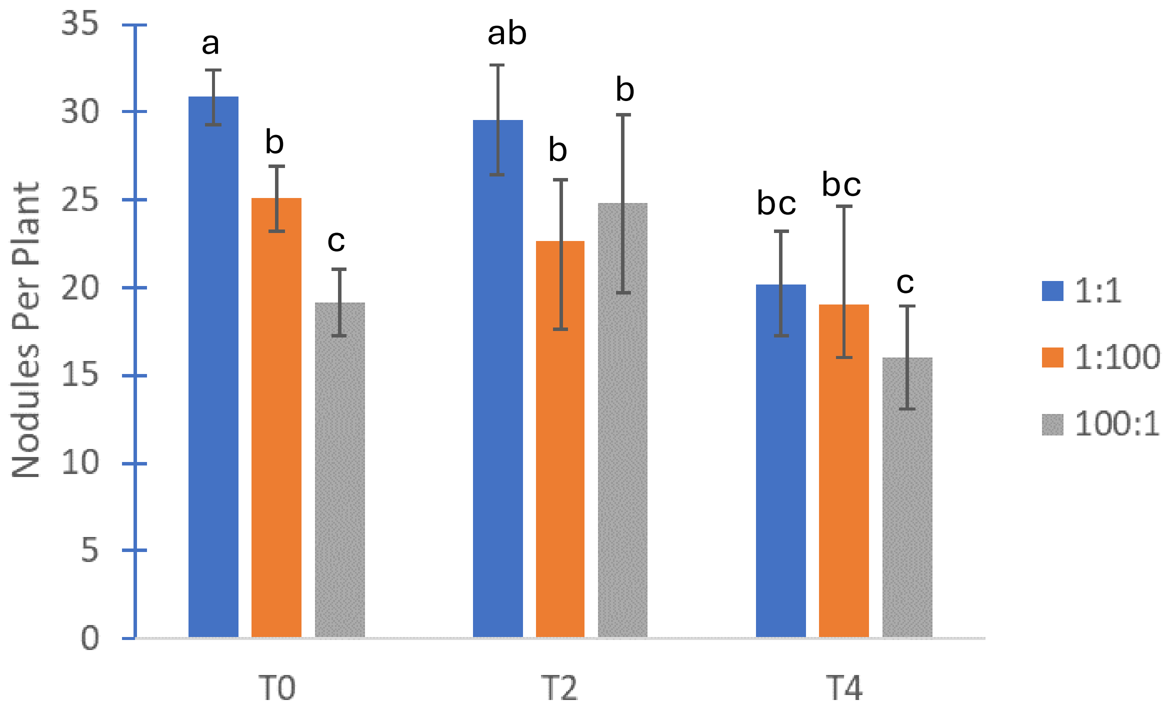

Fig. S9: Average shoot dry mass per plant (grams) with standard error bars. Different letters above the bars indicate statistically significant differences based on Tukey's HSD test; bars sharing the same letter are not significantly different. Data were collected from 72 plants.

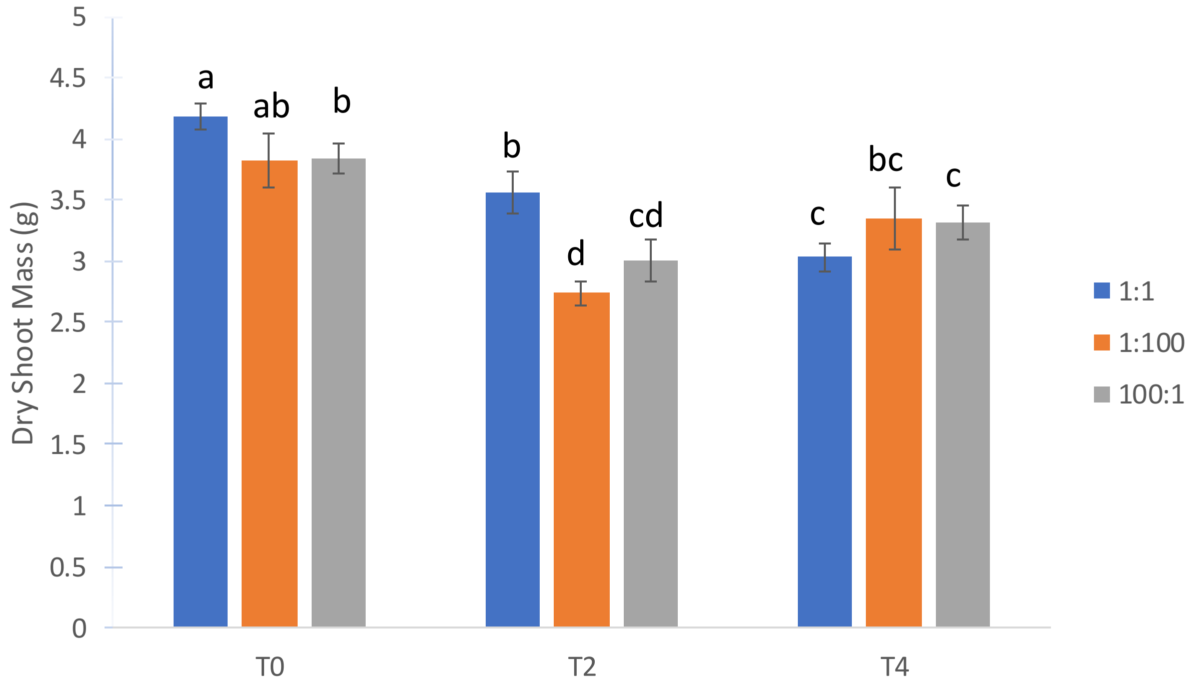

Fig. S10: Average root dry mass per plant (grams) with standard error bars. Different letters above the bars indicate statistically significant differences based on Tukey's HSD test; bars sharing the same letter are not significantly different. Data were collected from 72 plants.

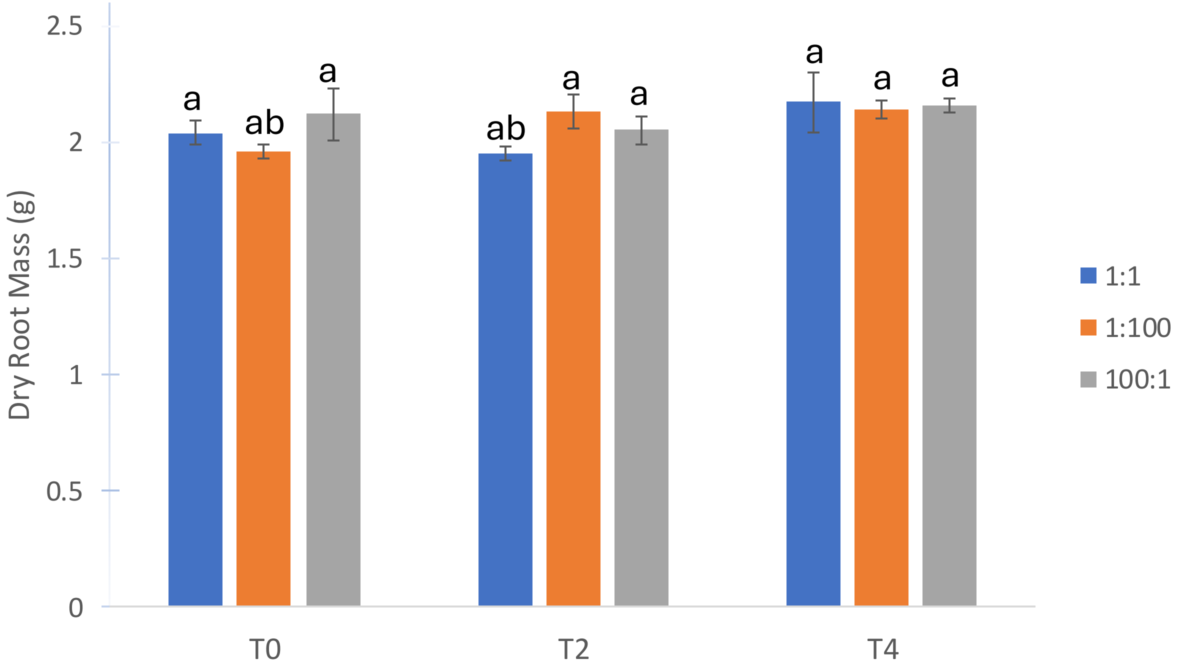

Supplement: Supplemental material — Tables S1 and S2; Fig. S1 to S10. [file aem.02489-25-s0001.pdf]
